# MODMatcher: Multi-Omics Data Matcher for Integrative Genomic Analysis

**DOI:** 10.1371/journal.pcbi.1003790

**Published:** 2014-08-14

**Authors:** Seungyeul Yoo, Tao Huang, Joshua D. Campbell, Eunjee Lee, Zhidong Tu, Mark W. Geraci, Charles A. Powell, Eric E. Schadt, Avrum Spira, Jun Zhu

**Affiliations:** 1 Icahn Institute of Genomics and Multiscale Biology, Icahn School of Medicine at Mount Sinai, New York, New York, United States of America; 2 Department of Genetics and Genomic Sciences, Icahn School of Medicine at Mount Sinai, New York, New York, United States of America; 3 Division of Computational Biomedicine, Department of Medicine, Boston University School of Medicine, Boston, Massachusetts, United States of America; 4 Division of Pulmonary Sciences and Critical Care Medicine, University of Colorado Denver, Aurora, Colorado, United States of America; 5 Division of Pulmonary, Critical Care and Sleep Medicine, Icahn School of Medicine at Mount Sinai, New York, New York, United States of America; University of Southern California, United States of America

## Abstract

Errors in sample annotation or labeling often occur in large-scale genetic or genomic studies and are difficult to avoid completely during data generation and management. For integrative genomic studies, it is critical to identify and correct these errors. Different types of genetic and genomic data are inter-connected by cis-regulations. On that basis, we developed a computational approach, Multi-Omics Data Matcher (MODMatcher), to identify and correct sample labeling errors in multiple types of molecular data, which can be used in further integrative analysis. Our results indicate that inspection of sample annotation and labeling error is an indispensable data quality assurance step. Applied to a large lung genomic study, MODMatcher increased statistically significant genetic associations and genomic correlations by more than two-fold. In a simulation study, MODMatcher provided more robust results by using three types of omics data than two types of omics data. We further demonstrate that MODMatcher can be broadly applied to large genomic data sets containing multiple types of omics data, such as The Cancer Genome Atlas (TCGA) data sets.

## Author Summary

Many human diseases are complex with multiple genetic and environmental causal factors interacting together to give rise to disease phenotypes. Such factors affect biological systems through many layers of regulations, including transcriptional and epigenetic regulation, and protein changes. To fully understand their molecular mechanisms, complex diseases are often studied in diverse dimensions including genetics (genotype variations by single nucleotide polymorphism (SNP) arrays or whole exome sequencing), transcriptomics, epigenetics, and proteomics. However, errors in sample annotation or labeling often occur in large-scale genetic and genomic studies and are difficult to avoid completely during data generation and management. Identifying and correcting these errors are critical for integrative genomic studies. In this study, we developed a computational approach, Multi-Omics Data Matcher (MODMatcher), to identify and correct sample labeling errors based on multiple types of molecular data before further integrative analysis. Our results indicate that signals increased more than 100% after correction of sample labeling errors in a large lung genomic study. Our method can be broadly applied to large genomic data sets with multiple types of omics data, such as TCGA (The Cancer Genome Atlas) data sets.

## Introduction

Cells employ multiple levels of regulation that enable them to respond to genetic, epigenetic, genomic, and environmental perturbations. With advances in high-throughput technologies, comprehensive data sets have been generated to measure multiple aspects of biological regulation, such as genetics, transcriptomics, metabolomics, glycomics, and proteomics. To elucidate the complexity of cell regulation, diverse types of data from these different technologies must be integrated.

Sample errors, including sample swapping, mis-labeling, and improper data entry are inevitable during large-scale data generation. Some of these errors can be detected during quality control (QC) on each type of data; however, others are more elusive and may affect integrative data analysis, depending on the integration methods used. In some integrative analyses, signature sets are first defined by each data type individually, for example signatures for gene expression, methylation, or copy number variation (CNV). Then, the signatures are overlapped to identify high-confidence changes [1]. In such analyses, potential sample inconsistencies may have a limited effect on results. For example, assume that samples A and B are swapped in gene expression data. If both samples are involved in the same subgroup (e.g., normal control or disease), the derived signatures will not be affected by the sample mis-labeling error. In other integrative analyses, such as the genetic gene expression studies [2], [3], in which the aim is to discover how DNA variations or single nucleotide polymorphisms (SNPs) regulate gene expression changes, sample errors could have a larger effect. In one study, mis-matching of 20% of samples between genotype and gene expression data decreased the number of cis-eSNPs by 70% [4].

To fully understand biological systems, it is necessary to elucidate how genetic and epigenetic perturbations lead to transcriptomic and proteomic changes, which in turn contribute to the disease phenotype. Simultaneously considering different types of biological data can result a better understanding of biological systems [2], [5]–[8].

With recent advances in high-throughput technologies, multiple layers of molecular phenotypes have been measured in the same sample for comprehensive survey of biological systems. To maximally utilize these data, it is necessary to properly match different types of data pertaining to the same sample or individual before integrative analyses. Here we present a sample mapping procedure called Multi-Omics Data matcher (MODMatcher), which not only identifies mis-matched omics profile pairs, but also properly assigns them to the correct samples based on other omics data. We applied MODMatcher to two large-scale public multi-omics datasets: one from the Lung Genomic Research Consortium (LGRC) and one from The Cancer Genome Atlas (TCGA). In both cases, adjustment for mis-matched samples improved data consistency and increased statistic power to identify biological regulations. All software programs and scripts are available at http://research.mssm.edu/integrative-network-biology/Software.html.

## Results

### Application to LGRC data

**Figure 1:**
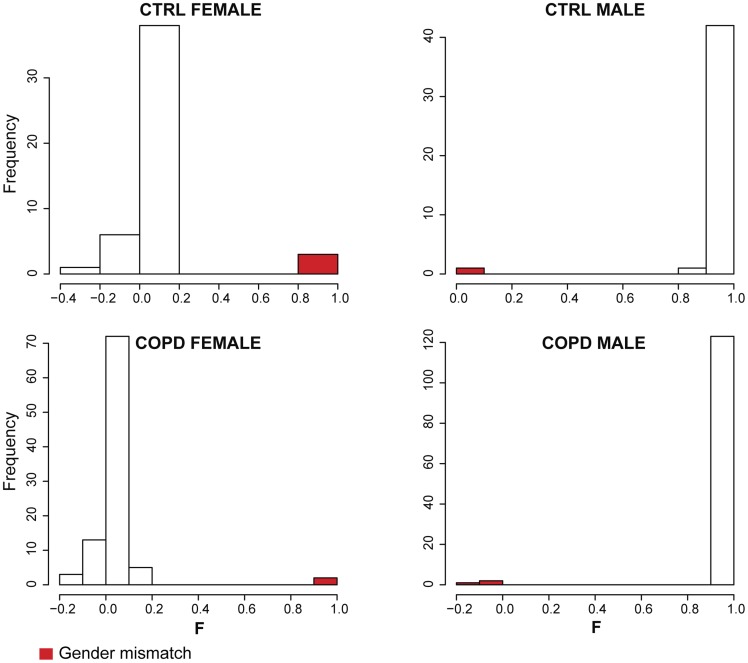
Gender prediction based on genotype data. The inbreeding coefficient F, the X chromosome heterozygosity rate, is used to infer the gender of samples. F is around 0 in most female samples and around 1 in most male samples. For 9 samples, the inferred genders were inconsistent with clinically annotated genders (error rate 3.5%).

**Figure 2:**
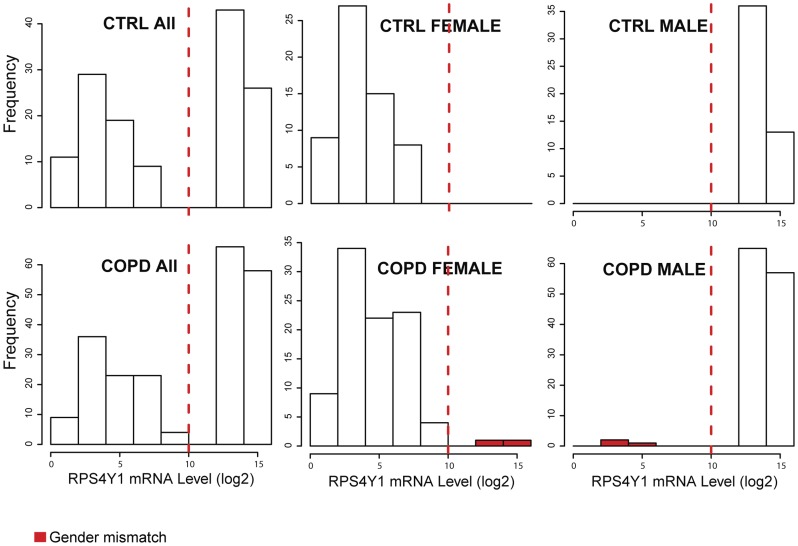
Gender prediction based on expression of the Y-chromosome specific gene *RPS4Y1*. The log2 transformed values of *RPS4Y1* expression level are clearly separated between male and female samples both in CTRL and patients with COPD (>10 in male samples and <10 in female samples). There were no gender mismatched samples in the CTRL and 5 mismatched samples (2 in females and 3 in males) in the COPD set (error rate of 1.5%).

**Figure 3:**
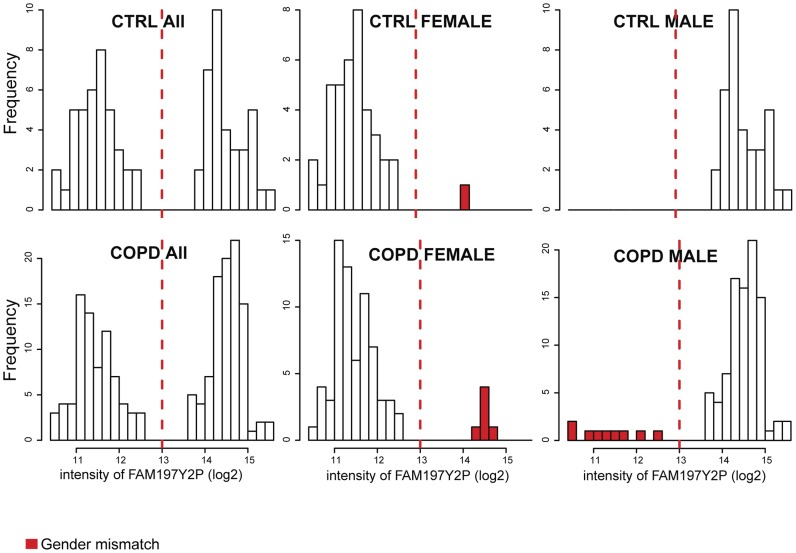
Gender prediction based on methylation intensity. The raw intensity of a Y-chromosome methyl probe corresponding to *FAM197Y2P* is clearly different between genders. One error was identified in the CTRL and 15 errors were identified in the COPD set (6 in females, 9 in males) (error rate of 6.4%).

The LGRC is a consortium for studying chronic lung diseases including chronic obstructive pulmonary disease (COPD). Clinical information and gene expression and methylation profiling data were obtained from the LGRC data portal (http://www.lung-genomics.org). Genotype data was provided by the LGRC consortium. The data set consists of gene expression profiles of lung tissues from 219 patients with COPD and 108 non-disease controls (CTRL), and methylation profiles of lung tissues from 173 COPD patients and 76 controls. First, the gender of each sample was inferred based on three types of data and compared to the gender annotated in clinical data. There was no ambiguity in gender prediction based on each individual type of data; the molecular profiles of different genders were clearly separated (Figures 1–3). However, we identified several mismatches between the predicted genders based on omics data and the clinically annotated genders. Among genders predicted by X-chromosome heterozygosity, we detected 4 mismatches in CTRL and 5 in COPD samples, corresponding to a mismatch error rate of 3.5% (9/256) for SNP genotype profiles (Figure 1). While there was no gender mismatch in CTRL samples, as judged by the expression level of Y-chromosome specific gene *RPS4Y1*, we detected 5 gender mismatches in COPD, corresponding to a mismatch error rate of 1.5% (5/327) for gene expression profiles (Figure 2). Among genders predicted from the intensity of the Y-chromosome specific methyl probe close to *FAM197Y2P* (see Methods), we found 1 gender inconsistency in CTRL samples and 15 in COPD samples, corresponding to a mismatch error rate of 6.4% (16/249) for methylation profiles (Figure 3). Overall, for 21 unique individuals (Table S1), the gender information inferred from different sources did not match either with one another or with clinical annotation, indicating sample alignment problems. According to the error rate of gender mismatches, gene expression profiling data was least likely to be mis-labeled, and methylation profiling data was most likely to be mis-labeled in the LRGC data set.

**Figure 4:**
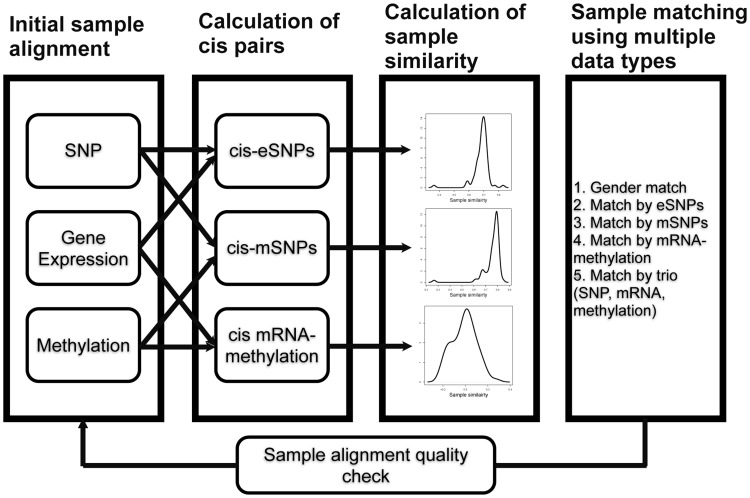
Sample alignment with MODMatcher. Initial labels of samples are used to determine cis pairs, which are then used to calculate similarity scores. Based on the similarity scores determined with three data types, the molecular data are matched with each other (1) by gender, (2) by cis-eSNPs, (3) by cis-mSNPs, (4) by cis mRNA-methylation pairs, and (5) by all trio mapping. Then, updated sample pairs are used to calculate new cis pairs for another round of alignment. Rounds of alignment are repeated until there are no further changes.

**Figure 5:**
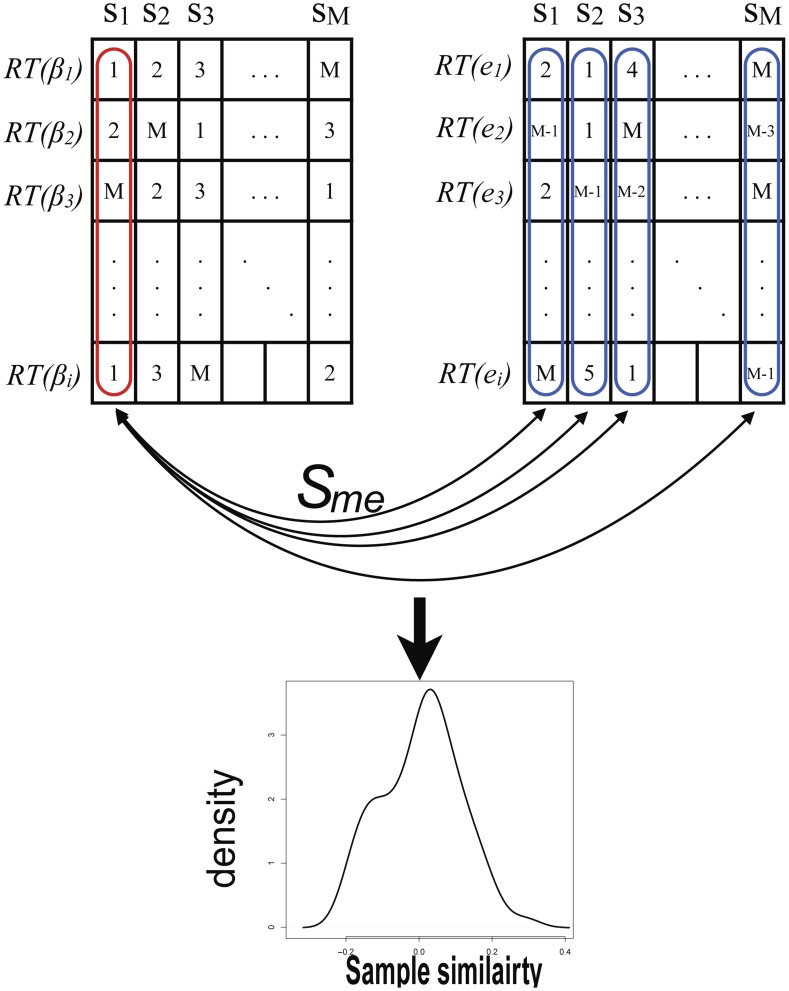
Sample similarity measurement based on cis methylation-mRNA pairs. After cis methylation-mRNA pairs are identified, the methylation and gene expression levels were rank-transformed. In this figure, there are M samples and *i* cis pairs. Then Pearson correlation is calculated and used as sample similarity, 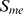, between one methylation profile and all gene expression profiles. If both methylation and gene expression profiles are from the same individual, self-self correlation coefficient is expected to be significantly higher than correlation coefficients with other samples.

**Figure 6:**
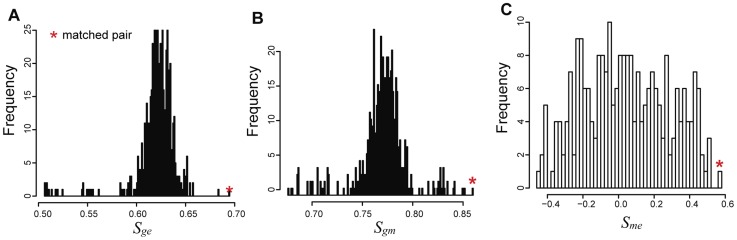
Distribution of similarity scores. (A) The distribution of similarity scores of one profile versus all profiles of other omics data type based on cis-eQTLs. (B) The distribution of similarity scores of one profile versus all profiles of other data type based on cis-mQTLs. (C) The distribution of similarity scores of one profile versus all profiles of other data type based on cis mRNA-methylation pairs. If two profiles pertain to the same sample (self-consistent), their similarity scores (red stars) are expected to be higher than those of cross-matched pairs.

Next, we iteratively matched SNP, gene expression, and methylation profiles using multi-omics identity similarity scores (Figure 4). We started with three sets of profile pairs with consistent inferred gender information: 179 pairs (50 CTRL and 129 COPD) for genotype and gene expression data, 182 pairs (51 CTRL 131 COPD) for genotype and methylation data, and 209 pairs (61 CTRL and 148 COPD) for methylation and gene expression profiling data. Cis regulation pairs (i.e. cis-eSNPs, cis-mSNPs, and cis methy-mRNA probes) were identified separately for CTRL and COPD samples. Sample identity similarity scores 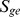, 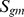, and 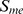 based on identified cis regulation pairs were calculated for all possible profile pairs. 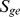 and 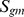 were calculated from the distance between predicted and measured SNP genotypes. 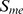 was measured by correlation of rank-transformed methylation and gene expression levels in samples (Figure 5, see Methods). The similarity scores for matched profiles were 3.8, 3.2, and 1.8 standard deviations better than the mean similarity scores for 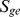, 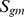, and 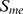, respectively (Figure 6A–C). Thus, SNP-mRNA sample matches were more reliable than SNP-methylation or methylation-mRNA sample matches, perhaps because methylation data tends to be noisy due to intrinsic technical design [9], [10]. Based on the gender-matching results, methylation profiles have a higher mis-label rate than other profile data, also contributing to the uncertainties of sample matching of methylation profiles.

Next, we determined whether mis-aligned samples could be matched with other unmatched samples by reciprocal best matching, based on one type of identity similarity score. In other words, we tested whether mis-aligned genotype profile *G_i_* had the highest similarity with an unmatched mRNA profile *E_j_* among all mRNA profiles, and the unmatched mRNA profile *E_j_* had the highest similarity with *G_i_* among all genotype profiles as well. For the sample pair with a reciprocal best match, sample labels can be updated by comparison with mapping results based on other identity similarities.

When all three types of data are available, the source of any sample labeling errors can be identified. It is also possible to remove or identify additional matched profiles that may be ambiguous as judged from 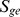, 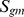, or 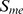 alone. Since cis-eSNPs pairs provided the best alignment signal, we started with sample matching by cis-eQTL. Then, samples were further matched by cis-mQTL and mRNA-methylation. For the SNP-mRNA profile match, we tested whether there was a methylation profile that matches well with both SNP and mRNA profiles in the matched pair.

**Figure 7:**
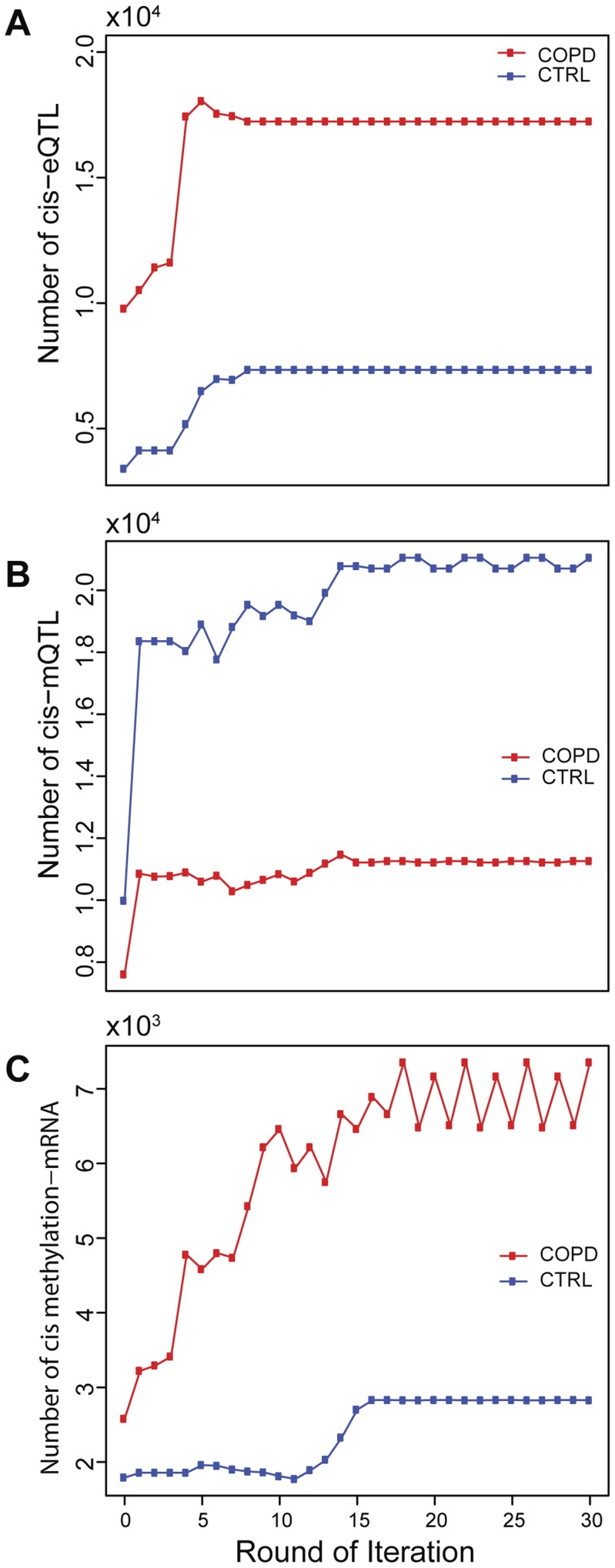
Assessment of sample alignment quality. The number of cis pairs is counted after each round of alignment. The number of cis pairs increased markedly after alignment in both the CTRL and COPD sets. The exact numbers of cis-pairs are listed in Table S2. A) cis-eQTLs. B) cis-mQTLs. C) cis mRNA-methylation pairs.

After each round of sample matching, the quality of sample alignment was assessed by counting the number cis pairs identified. For all pairs among these three data types, sample mapping correction significantly increased numbers of cis pairs identified (Figure 7). The number of cis-eSNPs stabilized within the first 5 rounds (Figure 7A). However, the number of cis-mSNP pairs stabilized in much later rounds (about 15–17), as expected because of the higher mis-label error rate and greater noise in the methylation data. Nonetheless, the numbers of cis-pairs involving methylation profiles increased substantially with the improved sample matching (Figure 7B and 7C). In COPD samples, the number of cis-eSNPs increased by ∼100% and the number of cis mRNA-methylation pairs increased by ∼200%. Consistently, fewer cis pairs were identified in the CTRL data set than in the COPD data set. This difference likely reflects disease biology. Although there were fewer CTRL than COPD samples and thus less statistical power, the trend of difference was the same when we sampled equal numbers of COPD samples to CTRL samples (Figure S1).

**Figure 8:**
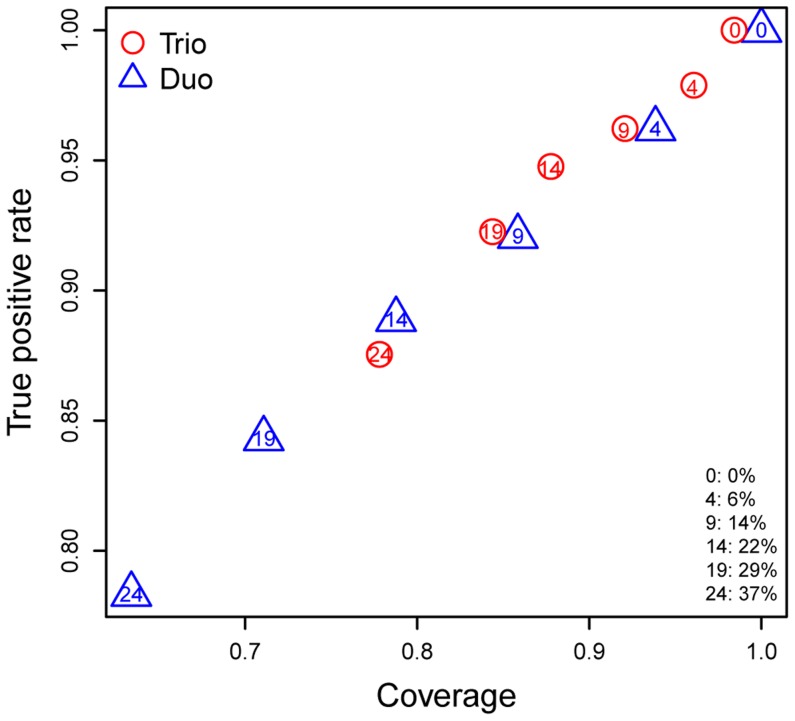
Comparison of sample alignment procedures based on three or two data types in simulated datasets. A total 65 COPD samples with all three types of data (gene expression, genotype, and methylation) were used. The mis-labeling error rates were fixed at 3% between gene expression and genotypes. The number of mis-aligned pairs was varied from 0 to 24 (corresponding error rate, 0% to 37%). Two sample alignment procedures were applied to the simulated data sets and final aligned pairs were compared with the true alignment. Triangles, duo-alignment results; circles, trio-alignment results. Numbers inside triangles or circles indicate the number of mis-aligned samples in each simulation. Coverage is defined as the number of correctly aligned pairs divided by 65 (the number of original pairs). The true positive rate is defined as the number of correctly aligned pairs divided by all aligned pairs.

Using a series of simulated data sets, we demonstrated that trio alignment (considering three types of data simultaneously) resulted in better alignment than duo alignments (considering two types of data at a time) combined. From the sample alignment of the LGRC data as describe above, we identified 76 COPD samples with aligned genotype, gene expression and methylation profiles. Among these 76 samples, only 65 could be correctly matched when individual similarity scores such as cis mRNA-methylation pairs were used. For a fair comparison of trio and duo alignment, we simulated sample labeling errors by randomly assigning sample labels using only these 65 COPD samples. As in the empirical data, we kept low error rates in SNP and gene expression profiling data. We increased the number of mis-labeled methylation profiles from 0 to 24 (corresponding error rate 0% to 37%). At each error rate, we simulated 5 independent data sets and used the average for comparison. In both of duo and trio alignment, all three data types were used but in different ways. In duo alignment, we identified the sample pairs from each pair of data types independently and summarized them to have final pairs. For an example, a methylation profile can be matched with an mRNA profile directly based on the identity similarity score 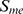 or through a chain of matches, in which the methylation profile is matched to an SNP profile which matches the mRNA profile. In trio alignment, there is an additional three-way identity similarity score that considers all three data types simultaneously (as described in Methods). Both trio and duo alignment identified mis-matches and improved data quality. However, trio alignment was more robust and superior, especially when mis-labeling error rates were high (Figure 8). Trio alignment recovered more samples pairs and predicted sample pairs more accurately than alignments considering similarity scores independently. In trio alignment, the additional data type provided more bridging information for matching mis-aligned samples pairs. Thus, at the same mis-labeling error rate, trio alignment yielded a higher true positive rate and better coverage (Figure 8). As error rates increased, the benefit of using trio alignment became clearer. Thus, in correcting sample mis-matches, sample alignment considering three types of data simultaneously in sample alignment may have advantages over combining three independent duo-alignments. These simulation results confirm that sample alignment using multi-omics data is a critical QC step. Alignment that considers three types of omics data simultaneously is strongly recommended if applicable. Nevertheless, duo alignment is still useful for identifying and correcting mis-aligned pairs.

### Application to TCGA data

#### 1) TCGA BRCA samples

**Figure 9:**
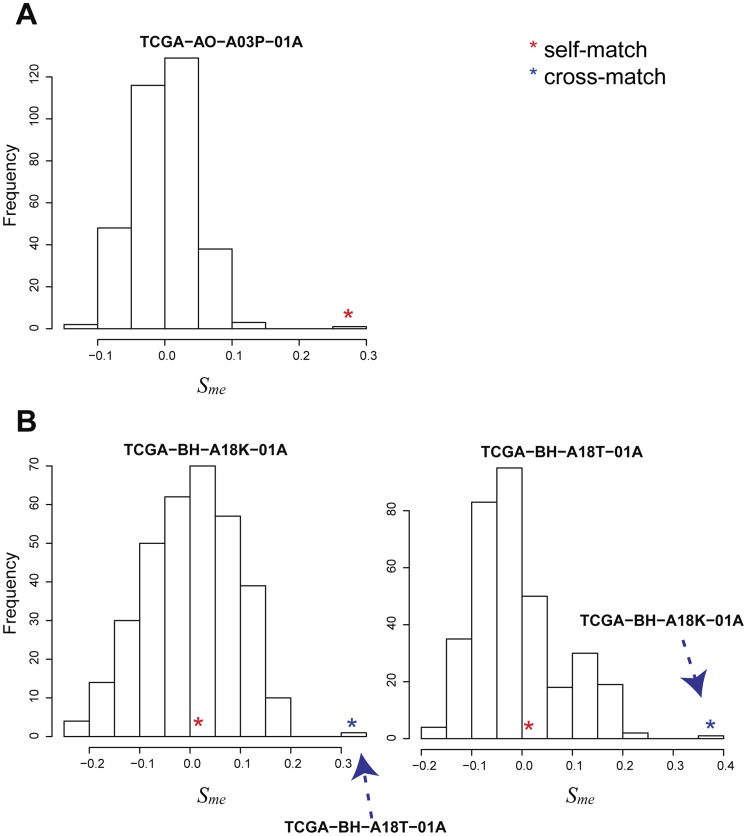
Examples of sample alignment in the TCGA BRCA data set. (A) A similarity score distribution of a correctly labeled profile. The red star indicates the similarity score between self-matched profile pairs (gene expression and methylation data profiles are labeled as pertaining to the same sample). (B) Similarity scores of self-matched pairs (red stars) between gene expression and methylation profiles for two samples are lower than the similarity scores of cross-matched pairs (blue stars).

The same sample alignment approach was applied to another publically available dataset, TCGA breast cancer samples. There were 317 tumor samples and 20 adjacent normal samples with both gene expression and methylation profiles (Table 1). Genders of samples were inferred from molecular markers in gene expression and methylation profiles. We detected one tumor sample whose predicted gender was inconsistent based on gene expression and methylation profiles. After removal of the gender mismatched sample, cis methylation-mRNA probe pairs were redefined for both normal and tumor samples. At p-value<0.01, 9195 pairs were identified for the tumor (FDR<0.02 based on permutation tests) and 537 pairs for normal samples (FDR<0.35). The identity similarity score 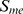 based on these cis probe pairs were normally distributed; one outlier had a higher similarity score (red star in Figure 9A), indicating a match of profiles of the same patient. There were 8 mis-aligned profile pairs among the tumor samples. Three profile pairs were matched by reciprocal mapping. Two of them, “TCGA-BH-A18**K**-01”, and “TCGA-BH-A18**T**-01”, were cross-aligned to each other in methylation and gene expression profiles (Figure 9B). Interestingly, the barcodes of two samples had only one difference (K vs. T), suggesting a sample swap in either the mRNA or methylation profiles. Further comparison with miRNA profiles of these tumor samples suggested that the swap was in the mRNA profiles (Figure S2). The updated sample alignment resulted in more cis pairs (9252 at p-value<0.01) and also stronger statistical p-values for the cis-correlations. For example, the p-value for the cis correlation of methylation and gene expression levels of *TMEM139* was 1.6×10^−67^ before alignment and 3.8×10^−74^ after alignment.

**Table 1:** Profile pairs used in TCGA dataset.

Data type pairs	BRCA	GBM
**CNV-mRNA**	165 tumor, 13 normal	470 tumor
**CNV-methylation**	149 tumor, 0 normal	294 tumor
**mRNA-methylation**	317 tumor, 20 normal	221 tumor

#### 2) TCGA GBM samples

**Figure 10:**
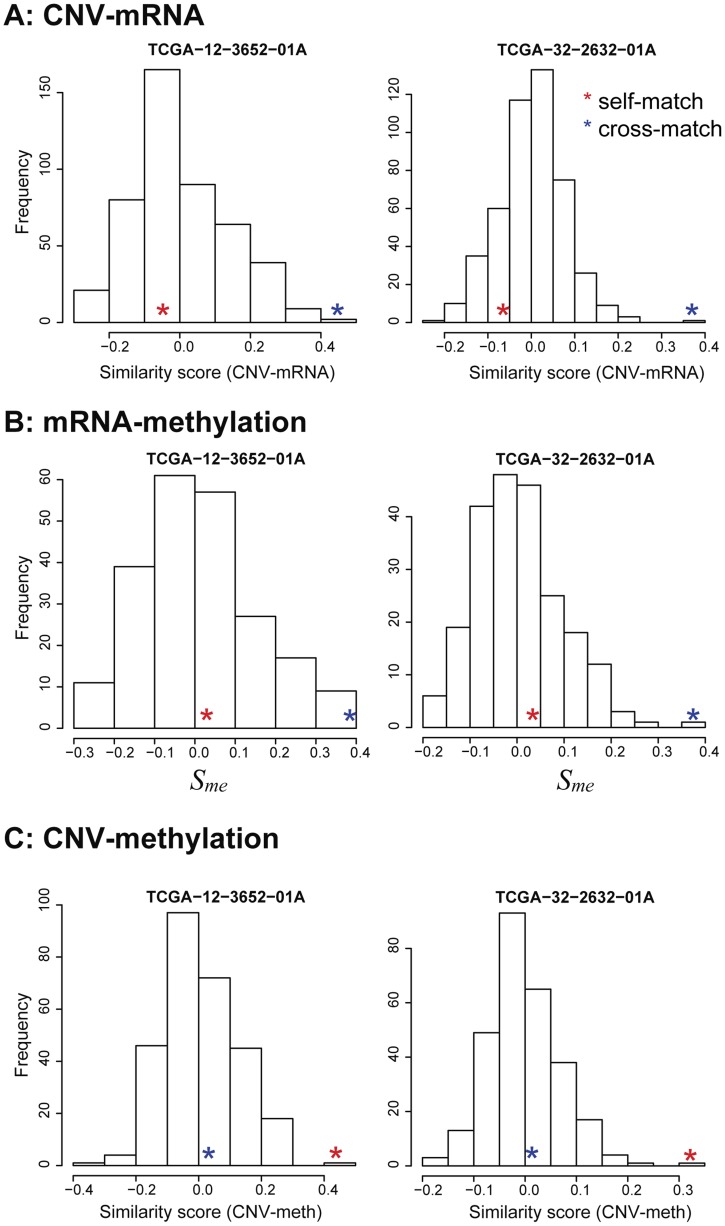
Identification of potential source of mis-labeling error by trio alignment in TCGA GBM samples. (A) The two GBM tumor samples were cross-matched between CNV and gene expression profiles. Red stars, similarity scores of self-matched pairs; blue stars, similarity scores of cross-matched profiles. (B) The two samples were also cross-matched between gene expression and methylation profiles. (C) Sample labels were consistent between CNV and methylation profiles. These results together indicate that the sample labeling error lay in the gene expression profiles.

TCGA glioblastoma multiforme (GBM) is the first cancer data set in TCGA consisting of CNV, gene expression, and methylation profiles. There were 470 GBM tumor samples with both CNV and mRNA profiles. We identified 24 mis-aligned profile pairs. Two of them were cross-aligned between CNV and gene expression profiles (TCGA-32-2632-01A, and TCGA-12-3652-01A) (Figure 10A). When we aligned methylation and gene expression profiles based on the identity similarity score 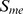 calculated by using cis methylation-mRNA pairs, they were cross-aligned to each other as well, indicating that the labels of mRNA profiles are problematic (Figure 10B). Additionally, the two samples were self-aligned between CNV and methylation profiles (Figure 10C). These findings indicate that the sample labels of the two mRNA profiles were swapped. This example shows how sample alignment using three different molecular data sets can be useful for both correcting alignment errors in sample pairs and identifying the source of the errors.

## Discussion

In large-scale genetic and genomic studies, errors in sample annotation or labeling are common and difficult to avoid completely. Identifying and correcting these errors is critical for statistical analysis, especially for integrative analysis. In this study, we introduce an iterative computational procedure, MODMatcher, that uses multiple types of molecular data (e.g., genotype, CNV, gene expression, and methylation profiles) for sample alignment by using cis regulation pairs of each pair of data types to calculate sample identity similarity scores. When applied to two large public data sets, LGRC and TCGA, MODMatcher not only identified mis-aligned profile pairs but also corrected and rescued mis-labeled samples. In a simulation study of COPD samples in the LGRC set, sample alignment with three types of data (trio matching) performed better than alignment with two types of data (duo matching). When applied to the GBM data set in TCGA, trio matching unambiguously identified the source of sample labeling errors. Thus, MODMatcher can rescue mis-aligned or mis-labeled samples to maximize statistical power in integrative analysis in large-scale genetic and genomic studies. Indeed, correction of mis-aligned samples increased both the number of cis pairs identified and the statistical significance.

Sample labeling errors are not unique to a few data sets, but are inevitable for any large data sets, despite intensive efforts in QCing each type of data individually. Our methods based on methylation profiles for gender inference and alignment with other omics profiles are novel and have not been included in standard data QC procedures. We applied our methylation-based gender inference method to more TCGA data sets and demonstrated that gender can be unambiguously inferred from methylation profiles (Figure S3). We identified 1, 4, 1, 2 gender mis-match errors in methylation profiles in data sets for colon adenocarcinoma (COAD), kidney renal papillary carcinoma (KIRC), acute myeloid leukemia (LAML), and lung adenocarcinoma (LUAD), respectively (Table S3). We also applied our methylation-gene expression profile matching method to additional TCGA data sets, COAD and lung squamous cell carcinoma (LUSC), and identified multiple mis-label errors (examples shown in Figure S4). Thus, checking sample alignment is a critical and necessary QC step before integrative analysis.

It is worth to note that the sample identity similarity scores, 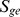, 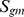, and 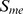, are calculated by using cis regulation pairs. Therefore, like the method of Westra et al. [4], MODMatcher depends on initial sample alignments to generate cis regulation pairs. However, MODMatcher is more robust and can tolerate extra noise, as shown in the simulation study. If the error rate of initial alignment is too high (e.g., >30% mis-alignment), we may not be able to identify enough cis-regulation pairs to accurately align samples on the basis of a single identity score. But based on three-way similarity, more accurate matching pairs can still be identified.

MODMatcher has several features not found in existing sample alignment methods such as MixupMapper [4]. First, we proposed novel methods for methylation profile based gender inference and sample alignment, and MODMatcher can be applied to diverse types of data, including genotype, gene expression, methylation, and CNV. MixupMapper can only be applied to genotype and gene expression data. Second, by using more than two types of omics profiles, MODMatcher can not only identify potential mis-labeled omics profile pairs, but also pinpoint which profiles in the pairs are mis-labeled (Figures 9 and 10), and do so more robustly than when only two types of omics profiles are used (Figure 8)

Even though MODMatcher is not designed for matching two types of omics profiles, it can be applied to data sets consisting of only two types of omics profiles. MixupMapper and MODMatcher can only be compared for their ability to match genotype and mRNA profiles. We applied MODMatcher to 8 data sets examined by MixupMapper (downloaded from http://genenetwork.nl/wordpress/mixupmapper/#additional) and compared alignment results based on the two methods (Table S4). MODMatcher results completely agreed with MixupMapper results in 6 of 8 data sets. For the two datasets in which the MODMatcher and MixupMapper results are different, we further assessed sample alignment quality by counting cis-eQLs identified based on the final matching results. We input final matching pairs identified by each method and their corresponding profiles to the same program, MatrixEQTL [11], to identify cis-eQTLs. In both cases, more cis-eQTLs were identified with MODMatcher results than with MixupMapper results (Table S5).

After labeling errors in omics profiles are identified and corrected by leveraging information from multiple omics profiles, the corrected profiles can be compared with clinical information to answer many biological questions, such as what genes' expression levels correlate with blood lipid level, and what genes' methylation levels correlate with survival of cancer patients. To accomplish these tasks, we assume that all clinical data are correct, which may not always be true. There could be errors in clinical data files, such as missing data, and row or column shifts. It is more challenging to identify and correct errors in clinical data files than it is to identify labeling errors in omics profiles. More research efforts are warranted for checking potential errors in the links between clinical data and omics profiles.

## Materials and Methods

Comprehensive data sets generally consist of clinical or phenotypic data and multiple types of high-throughput data. For example, the LGRC data set consists of clinical, genotype, gene expression, and methylation data. The TCGA tumor data sets consist of clinical, genotype, gene expression, CNV, methylation, miRNA, and protein array data. Our procedure is applicable to data sets with clinical data and at least two different types of omics data. Here we illustrate our procedure on data sets with clinical data and genetic, gene expression, and methylation data.

### Datasets

#### 1) LGRC data set

Clinical, gene expression and methylation data were obtained from the LGRC data portal (http://www.lung-genomics.org). The genotypic data was provided by the LGRC consortium. The gene expression data was generated with Agilent V2 human whole genome arrays. The processed mRNA array data was downloaded from the LGRC website. DNA methylation data was generated with Nimblegen 2.1M Whole-Genome Tiling Arrays. Raw DNA methylation data was downloaded from the LGRC website. The quality of each probe was compared with background probe signals, and probes with low quality were excluded from further analysis. Then DNA methylation level (β value) of each tiling probe was estimated with the CHARM method [9], [12]. We confirmed that the estimated methylation level for each sample is almost identical with the processed methylation level data from the LGRC website. There were gene expression arrays for lung tissues from 219 COPD patients and 108 non-disease controls (CTRL) and methylation arrays for lung tissues from 173 COPD patients and 76 controls.

#### 2) TCGA data set

Different types of clinical and molecular data of various cancers are publicly available at the TCGA data portal (https://tcga-data.nci.nih.gov/tcga/). To illustrate our sample alignment procedure, we selected BRCA (one of the newest cancer data sets) [13] and GBM (the oldest cancer data set) [14]. Gene expression for the GBM and BRCA data sets was measured with microarrays. Methylation profiles were measured with Illumina HumanMethylation27 BeadChips. CNV data were generated with Affymetrix Genome-Wide Human SNP Array 6.0. Bulk data on BRCA and GBM samples was downloaded and processed individually. Each type of data was normalized between samples by quantile normalization and adjusted for covariance (e.g., batch number, plate number, center ID, and source site ID of sample). Samples were initially matched according to their labels as shown in Table 1.

### Gender inference

Gender information is generally included in clinical data. We also inferred gender information from genotype, gene expression, and methylation profiling data.

The gender of samples can be predicted from X-chromosome heterozygosity rates determined with PLINK [15]. An individual is predicted to be male if the estimated inbreeding coefficient F is >0.8 and female if F<0.2 [16]. There were inconsistencies between gender inferred from genotype data and gender provided in clinical data for the LGRC samples (Figure 1).

Gene expression levels of Y-chromosome specific genes can also be used to reliably predict gender information. *RPS4Y1* (ribosomal protein S4, Y-linked 1) is highly expressed in male [17]. Its expression level can robustly classify samples into male and female [6]. Figure 2 shows gender mismatches between clinical and gene expression data in the LGRC data set.

Raw intensity data in methylation profiling was used to determine whether probes mapped to Y-chromosome DNA fragments can be used to classify samples into male and female. Raw intensities of probes representing the Y-chromosome specific genes *FAM197Y2P*, *TTTY15*, and *TBL1Y* were significantly associated with genders in the LGRC data set (t-test p-values = 3.25×10^−28^, 1.79×10^−27^, and 8.71×10^−26^, respectively). A methyl probe, “chrY:9994006”, representing *FAM197Y2P* is the best methyl probe for gender prediction and was used to classify samples in the LGRC data set into male and female. Figure 3 shows that a higher mismatch rate between clinical and methylation profiling data than other pairs of data matching in the LGRC data set (Table S1).

### cis pair mapping

#### 1) cis-eSNP mapping

An eSNP is a single nucleotide polymorphism (SNP) whose genotype associates with variation in the expression of a particular gene. If that gene and its corresponding eSNP are in proximity, the eSNP is called a cis-eSNP. Cis-eSNPs have been extensively studied for their association with disease risks [2], [6], [18], and have been used to infer sample genotypes from gene expression profiling data [4], [19]. To identify cis-eSNPs, we used an efficient eSNP mapping program, MatrixEQTL [11]. Assuming that genotype had an additive and linear effect on gene expression, we calculated the t statics for each SNP and gene expression pair to evaluate the significance of association. Cis-eSNPs are defined as SNPs within 1 Mb of the genome region of the associated genes. The FDR (False Discovery Rate) was estimated from p-values with the procedure of Benjamini and Hochberg [20].

After cis-eSNPs were identified, the genotype 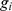 of the cis-eSNP for a particular sample 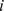 is inferred from the associated gene expression level 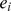 as follows. First, the mean gene expression level 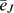 for each genotype 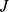 (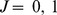 for haploid cells and 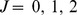 for diploid cells) is estimated by using all samples except sample *i*. Second, genotype at the cis-eSNP for sample *i* is inferred by comparing its gene expression level 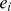 with the mean expression level of each genotype; the genotype whose mean is the closest to 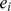 is assigned as the inferred genotype of sample *i* at the cis-eSNP location, noted as 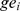.

Given sample genotypes measured by SNP array and inferred from cis-eSNPs, the sample identity similarity between the two genotypes is defined as 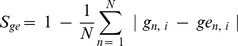, where 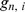 and 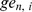 are the observed genotype based on the given sample labels (which may be incorrect due to sample mis-labeling) and the inferred genotype at the *n*th cis-eSNP for sample *i*, respectively, and *N* is the total number of cis-eSNPs.

#### 2) cis-mSNP mapping

Similar to eSNPs, genotypes of SNPs are also associated with DNA methylation patterns and are called mSNPs [21], [22]. To identify association between SNP genotype and methylation level, we used the SNP association mapping program MatrixEQTL [11], with input changed from gene expression profiles to DNA methylation profiles. Similarly, cis-mSNPs are defined as mSNPs within 1 Mb from the genomic regions of the associated methylation probes.

After cis-mSNPs are identified, the genotype 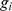 of the cis-mSNP for a particular sample *i* can be inferred from the associated probe methylation level 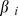 as follows. First, the mean probe methylation level 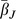 for each genotype 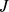 (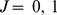 for haploid cells and 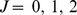 for diploid cells) is estimated by using all samples except sample 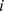. Second, genotype at the cis-mSNP for sample *i* is inferred by comparing its methylation level 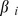 with 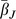 the mean methylation level of each genotype 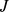; the genotype whose mean is the closest to 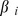 is assigned as the genotype of sample *i* at the cis-mSNP location, noted as 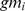.

Given sample genotypes measured by SNP and inferred from cis-mSNPs, the sample identify similarity between the two genotypes is defined as 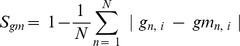, where 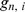 and 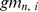 are observed and inferred genotype at the *n*th cis-mSNP for sample *i*, respectively, and *N* is the total number of cis-mSNPs.

#### 3) cis methylation-mRNA mapping

DNA methylation is a common epigenetic signal that regulates gene expression levels. Increased methylation at CpGs sites near gene promoter region is associated with gene repression [23], [24]. Transcript annotation of hg18 was fetched from UCSC database and further processed with the Bioconductor GenomicFeature package. Each methyl probe was mapped to a transcript whose starting site is within 10 Kb from the genomic position of the methyl probe. A methyl probe that is potentially mapped to multiple transcripts on the basis of the above criterion is assigned to the transcript whose start site is closest to the genomic position of the methyl probe. Methyl probes that can't be mapped to any transcript based on the above criterion were excluded from further analysis. To identify cis-regulation pairs, we calculated the Spearman correlation between the methylation level of a methyl probe and the expression level of the corresponding gene at p-value<0.01. If multiple methyl probes were mapped to the same genes, the probe with the best p-value was selected. Therefore, in subsequent analyses, there was at most a single cis methylation-mRNA pair for each gene. Thus, any potential bias driven by a single gene was avoided.

Before aligning methylation and mRNA profiling data, we rank transformed both gene expression and methylation profiling data for each methyl probe or gene expression probe as 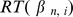 and 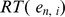, where 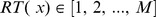 is the rank transformation function and 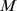 is the number of samples (Figure 5). Given a set of cis methylation-mRNA pairs 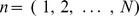, the sample identity similarity between the two types of data is defined as
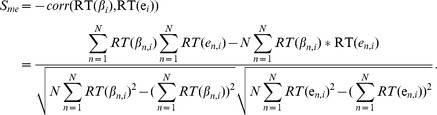



#### 4) cis CNV-mRNA (or methylation) mapping

Copy number variations (CNVs) of genome regions are commonly associated with diseases and may be inherited or occur by *de novo* mutations. Increasing the DNA copy number of a gene can increase its expression level. Instead of genotype, CNV data was aligned to gene expression and DNA methylation profiles in TCGA data sets. Cis regulation pairs and identity similarity scores were defined similarly as described above for methylation-mRNA pairs.

### Multi-omics data matching procedure

Multiple omics data surveying different molecular traits pertaining to the same set of samples were mapped according to the flow diagram in Figure 4. SNP genotype, gene expression, and methylation data are used for illustration purposes. Other types of data can be used as well. For example, CNV data was used instead of SNP data in the TCGA data sets. First, significant cis regulation (cis-eSNPs, cis-mSNPs, and cis methyl-mRNA) pairs were identified, and sample identity similarities were calculated based on these cis pairs as outlined above. Then, matches and mismatches between omics data were identified in the following steps (ordered by confidence of each test):

1. **Match by gender.** There is no ambiguity for the gender inferred from an omics profile. Any matched pair of omics profiles should have consistent gender information.
2. **Match by SNP-mRNA based identity similarity** 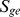 **.** After cis-eSNPs are identified, identity similarity 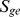 is calculated for all possible pairs of SNP-mRNA profiles based on the identified cis-eSNPs. The identity similarity 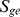 of SNP and gene expression profiles of the same individual is significantly higher than that of random pairs of profiles (Figure 6A). If the self similarity score 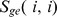 is within the top “n” similarity scores of all possible pairs reciprocally (the genotype profile 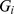 mapping to gene expression profiles and the gene expression profile 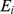 mapping to all genotype profiles), the pair of profiles is designated as correctly aligned. The “n” is ≤3 depending on the data set. To determine the value of “n”, we calculated the z-score of a genotype profile 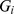 mapped to a gene expression profile 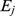 and *vice versa* as 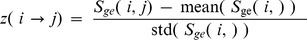 and 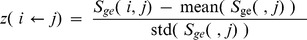, and then compared the z-score distribution of all top 1 similarity scores with the distribution of z-scores of all top “n” similarity scores. If the z-score distribution of all top 1 similarity scores is statistically different (t-test p-value<0.01) from the distribution of z-scores of all top 2 similarity scores, then “n” is set to 1. Otherwise, “n” is set to 2. In this fashion, we also compared the distributions of top 1 and top 3 similarity scores. For SNP-mRNA matching in the LGRC data set, top “n” was set as 1. For mis-aligned profiles, we further explored whether they could be matched with other unmatched samples by reciprocal matching, in which we determine whether a mis-aligned genotype profile *G_i_* has the highest similarity with an unmatched mRNA profile *E_j_* among all mRNA profiles, and the unmatched mRNA profile *E_j_* has the highest similarity with *G_i_* among all genotype profiles. If there is a reciprocal best match, then the SNP and mRNA profiles are linked and sample labels are updated by comparison with mapping results based on other identity similarities.
3. **Match by SNP-methylation based identity similarity** 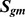 **.** After cis-mQTL are identified, the identity similarity score 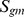 is calculated for all possible SNP and methylation profile pairs based on the set of identified cis-mSNPs. The identity similarity score 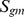 between SNPs and methylation profiles of the individual is higher than other random pairs (shown in Figure 6B). As above, if the self similarity score 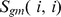 is within the top “n” similarity scores of all possible pairs, the pair of profiles is designated as correctly aligned. Top “n” was set to 3 in the LGRC data set. For mis-aligned profiles, we again further explored whether they could be matched with other unmatched samples by the reciprocal best matching procedure described above.
4. **Match by mRNA-methylation based identity similarity** 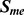 **.** After cis methylation-mRNA probes are identified, the identity similarity score 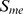 is calculated for all possible pairs of methylation-mRNA profiles based on the set of identified cis methylation–mRNA probes. The identity similarity score 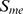 of methylation-mRNA probes pairs of the same sample is higher than random pairs (Figure 6C). If the self similarity score 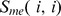 is within the top “n” similarity scores of all possible pairs, the pair of profiles is designated as correctly aligned. Top “n” was set to 3 in the LGRC data set similar as above. For mis-aligned profiles, we used the reciprocal best matching procedure described above to determine whether they could be matched with other unmatched samples.
5. **Match by trio (simultaneously considering** 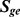 **,** 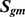 **, and** 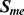 **).** For the samples with all three types of data available, the source of any sample label mis-matches can be identified (Figure 4). For example, if we identify a sample mapping between the gene expression profile of individual A and the methylation profile of individual B, it is difficult to know which profile data is mis-labeled or both. If the gene expression profile of individual A matches the SNP profile of individual A based on 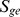 and the methylation profile of individual B is mapped with the SNP profiles of individual A, then it is certain that the methylation profile of individual B is mis-labeled. It is also possible to resolve matching conflicts and to identify additional matched profiles that may be ambiguous based on a single identity similarity score 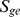, 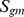, or 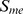 alone. For example, if data quality is low or the initial profile labeling error rate is high for methylation data, then 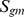 and 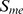 cannot be accurately calculated. If an SNP-mRNA sample match exists (SNP profile ***G*** *_i_* matches gene expression profile ***E*** *_j_*, then we can search whether there is a methylation profile ***M*** *_k_* that matches ***G*** *_i_* and ***E*** *_j_* by a three way identity similarity score as 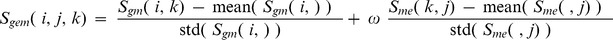, where *ω* is the weight of similarity 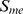 relative to 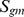. 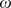 was set as 1.2 for the LGRC data set, reflecting the fact that the matching signal between genotype and methylation data is stronger than the matching signal between methylation and gene expression data. 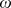 can be estimated as
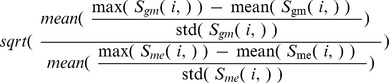
, which is the square root of the ratio of mean maximum z-scores of each profile under each similarity measurement. To declare methylation profile ***M*** *_k_* a match with ***G*** *_i_* and ***E*** *_j_*, both 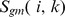 and 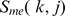 are required to be within top 3 among all possible similarity scores 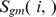 and 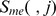, respectively, and 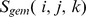 is ≥2.5.

After label mis-matches between different types of omics data are identified and sample labeling errors are corrected by comparing multiple identity similarity measurements, the quality of sample alignment is re-assessed by counting the numbers of cis regulation pairs according to the updated data annotation. We iterate this process until data annotations are stable.

## Supporting Information

Figure S1 Numbers of cis methyl-mRNA pairs in CTRL and COPD samples when equal numbers of samples were used.
(TIF)

Figure S2 The cross-aligned sample pair (TCGA-BH-A18T-01A and TCGA-BH-A18K-01A) identified by methylation-mRNA comparison was cross-aligned based on miRNA and mRNA comparison. Similarity scores based on cis miRNA-mRNA were around zero for the same labels but similarity scores for swapped pairs were the highest in both samples. Combined with the results shown in Figure 9B in main text, mRNA labeling for these two samples was likely to be problematic.
(TIF)

Figure S3 Gender prediction based on methylation probe intensity in 12 cancer types in the TCGA dataset. The raw intensity of a y-chromosome probe was estimated by summation of the methylated and unmethylated channel. The methyl probe “cg20401529” corresponding to *PRKY* was used as a gender marker for Illumina HumanMethylation27 Beadarray. For PRAD, for which only the HM450 platform is available, the methyl probe “cg04042030” corresponding to *TBL1Y* was used. Red, sample predicted to be female; blue, sample predicted to be male. The consistency between clinical and predicted gender is reported in Table S3.
(TIF)

Figure S4 Examples of mis-aligned pairs of mRNA and methylation profiles in the TCGA COAD and LUSC datasets. The similarity score for the same sample pairs based on cis methylation-mRNA pairs was not significantly higher than that of other pairs, indicating mis-alignment.
(TIF)

Table S1 Samples of mismatched gender information between clinical annotation and inference from multi-omics data (genotype, mRNA, and methylation profiles). Red ones are mismatched with respect to clinical annotation.
(XLSX)

Table S2 Numbers of cis pairs in each round of alignment corresponding Figure 7.
(XLSX)

Table S3 Gender inference based on methylation probe intensity in multiple cancer data sets in TCGA. The prediction (Figure S3) is compared with the clinically annotated gender. There are gender mismatched samples in four datasets, COAD, KIRC, LAML, and LUAD.
(XLSX)

Table S4 Comparison of MODMatcher and MixupMapper sample alignments between SNP and mRNA profiles on the same dataset. MODMatcher was applied into 8 dataset including genotype and mRNA profiles examined by MixupMapper. MODMatcher and MixupMapper generated the same result for 6 dataset and there are small differences in for other two dataset.
(XLSX)

Table S5 Qualities of sample matching results based on MixupMapper and MODMatcher. For the two datasets (Choy CHB+JPT and Choy YRI) where ModMatcher and MixupMapper results were different as shown in Table S4, the numbers of cis-eQTL pairs identified by each alignment method were compared and MODMatcher identified more cis-eQTLs in both dataset. Sample pairs identified in the two data sets by MODMatcher are listed in Tables S6 and S7.
(XLSX)

Table S6 Samples pairs in the Choy CHB+JPT data set identified by MODMatcher.
(XLSX)

Table S7 Samples pairs in the Choy YRI data set identified by MODMatcher.
(XLSX)
