# Supplementary figures and images for: MODMatcher: Multi-Omics Data Matcher for Integrative Genomic Analysis
Source: PLoS Comput Biol. 2014 Aug 14;10(8):e1003790. doi: 10.1371/journal.pcbi.1003790 (PMC4133046; doi:10.1371/journal.pcbi.1003790)

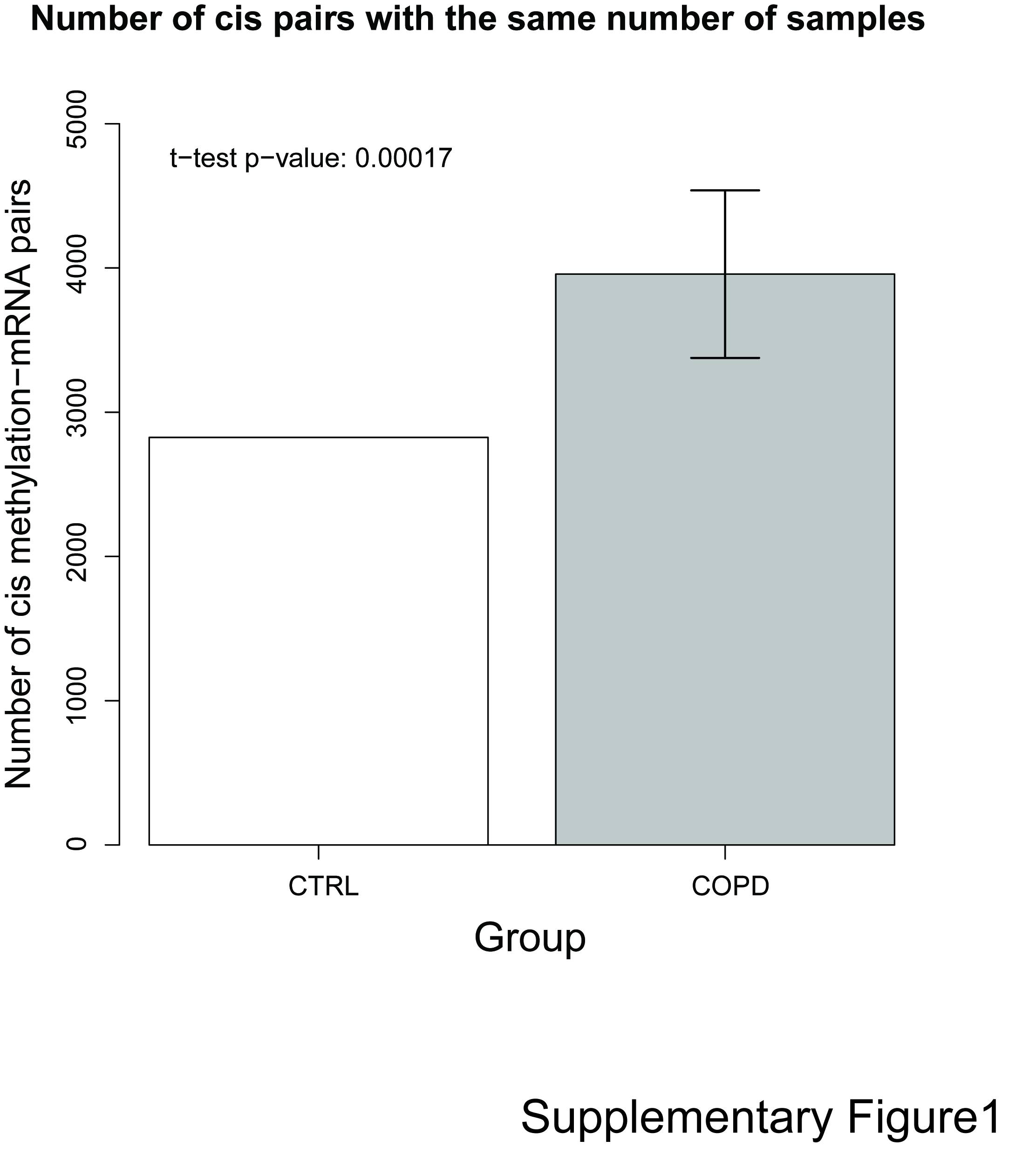

Supplement: Figure S1 — Numbers of cis methyl-mRNA pairs in CTRL and COPD samples when equal numbers of samples were used. (TIF) [file pcbi.1003790.s001.tif]

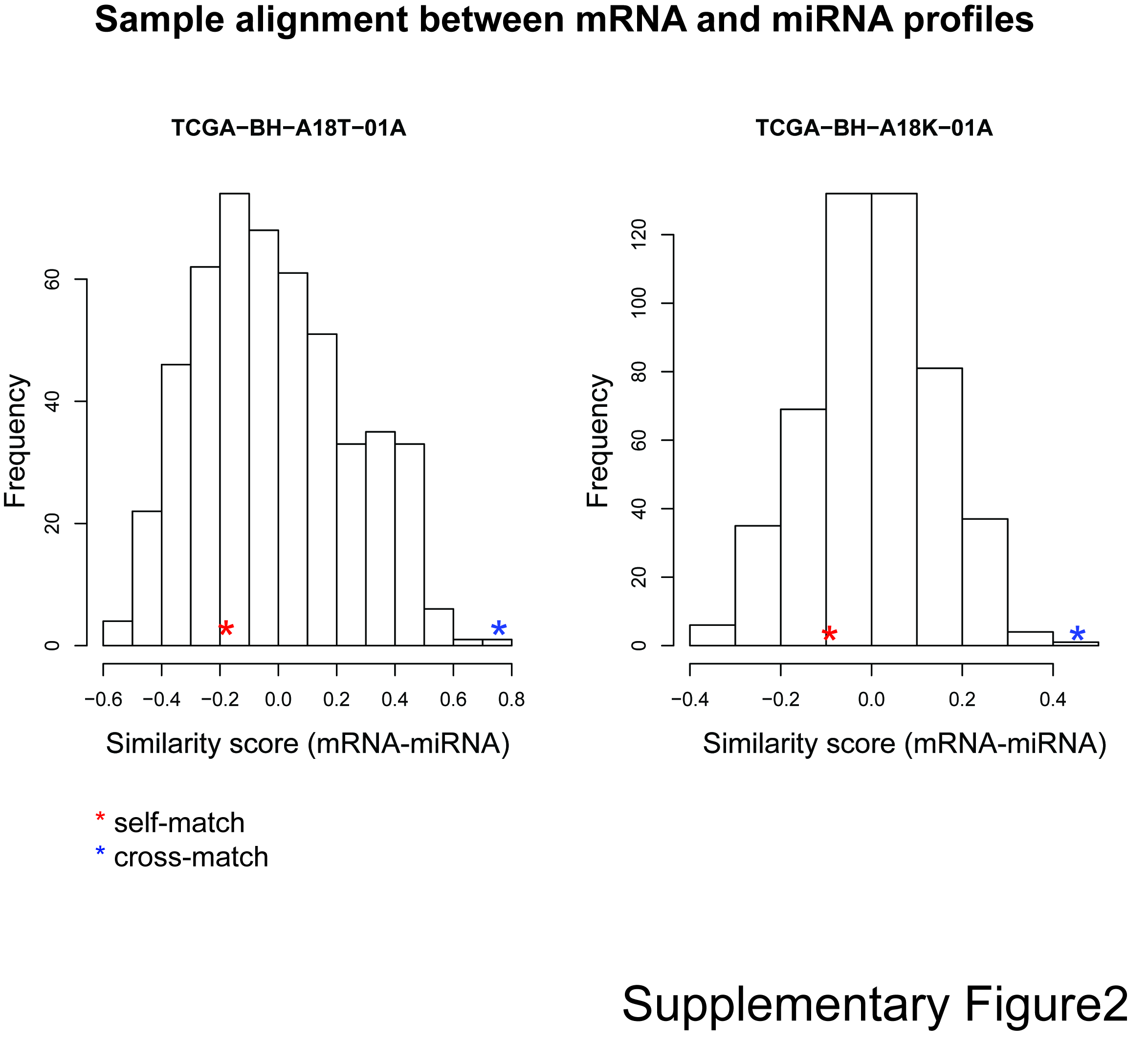

Supplement: Figure S2 — The cross-aligned sample pair (TCGA-BH-A18T-01A and TCGA-BH-A18K-01A) identified by methylation-mRNA comparison was cross-aligned based on miRNA and mRNA comparison. Similarity scores based on cis miRNA-mRNA were around zero for the same labels but similarity scores for swapped pairs were the highest in both samples. Combined with the results shown in Figure 9B in main text, mRNA labeling for these two samples was likely to be problematic. (TIF) [file pcbi.1003790.s002.tif]

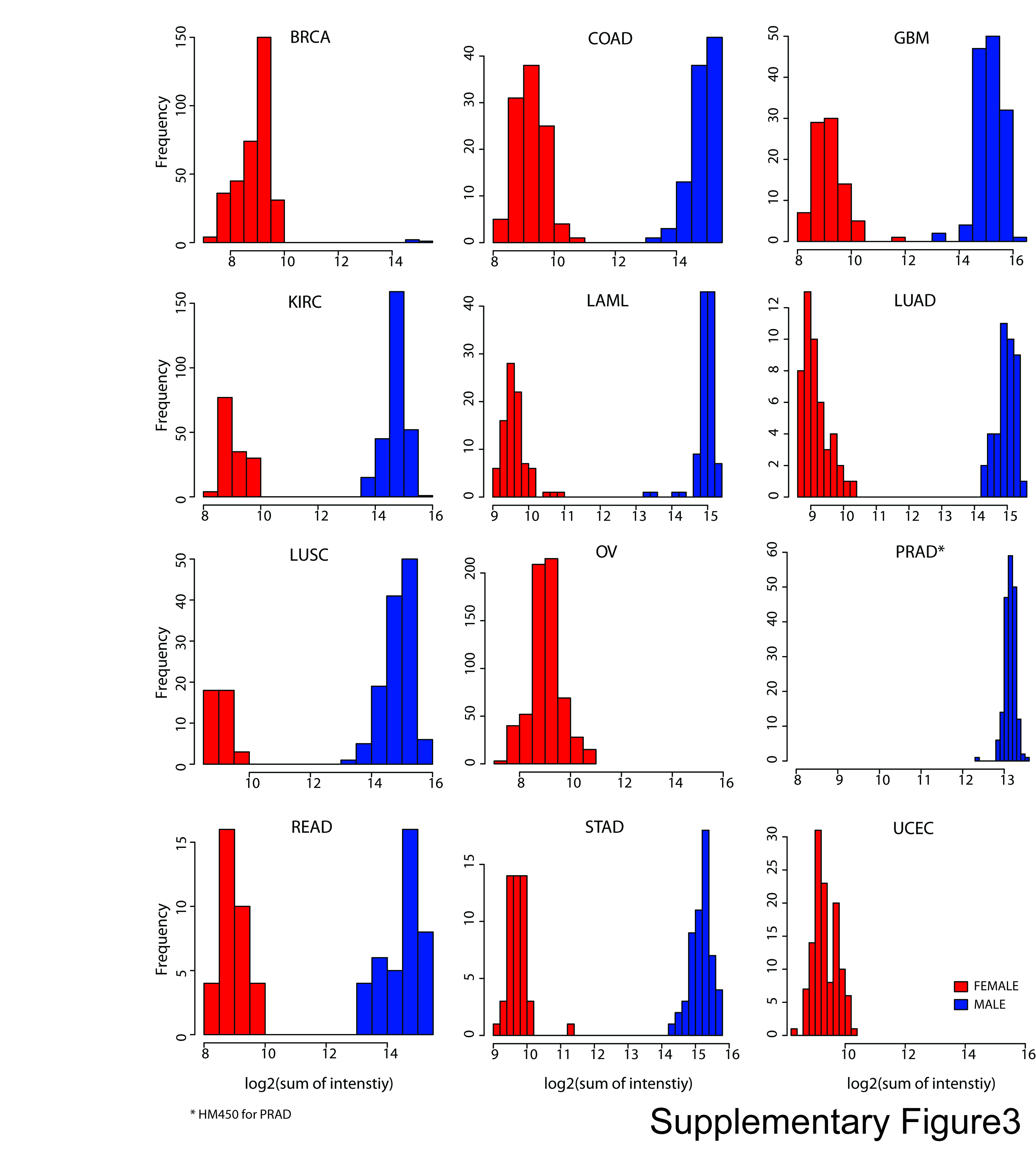

Supplement: Figure S3 — Gender prediction based on methylation probe intensity in 12 cancer types in the TCGA dataset. The raw intensity of a y-chromosome probe was estimated by summation of the methylated and unmethylated channel. The methyl probe “cg20401529” corresponding to PRKY was used as a gender marker for Illumina HumanMethylation27 Beadarray. For PRAD, for which only the HM450 platform is available, the methyl probe “cg04042030” corresponding to TBL1Y was used. Red, sample predicted to be female; blue, sample predicted to be male. The consistency between clinical and predicted gender is reported in Table S3. (TIF) [file pcbi.1003790.s003.tif]

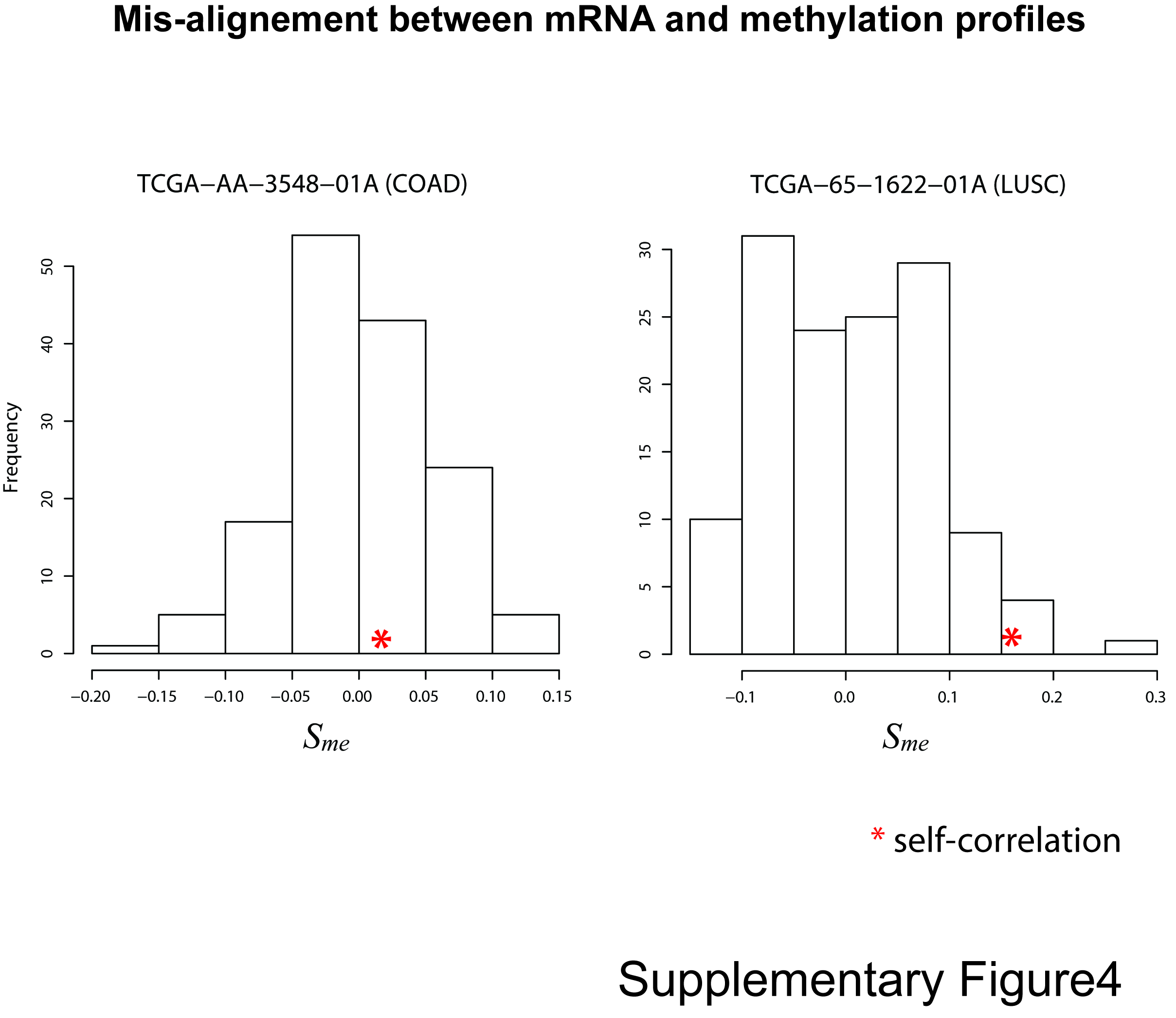

Supplement: Figure S4 — Examples of mis-aligned pairs of mRNA and methylation profiles in the TCGA COAD and LUSC datasets. The similarity score for the same sample pairs based on cis methylation-mRNA pairs was not significantly higher than that of other pairs, indicating mis-alignment. (TIF) [file pcbi.1003790.s004.tif]
